# The Thessaloniki ESHRE/ESGE consensus on diagnosis of female genital anomalies

**DOI:** 10.1007/s10397-015-0909-1

**Published:** 2015-11-04

**Authors:** Grigoris F. Grimbizis, Attilio Di Spiezio Sardo, Sotirios H. Saravelos, Stephan Gordts, Caterina Exacoustos, Dominique Van Schoubroeck, Carmina Bermejo, Nazar N. Amso, Geeta Nargund, Dirk Timmermann, Apostolos Athanasiadis, Sara Brucker, Carlo De Angelis, Marco Gergolet, Tin Chiu Li, Vasilios Tanos, Basil Tarlatzis, Roy Farquharson, Luca Gianaroli, Rudi Campo

**Affiliations:** Congenital Uterine Malformations (CONUTA) Common ESHRE/ESGE Working Group and Invited Experts, Leuven, Belgium; 1st Department of Obstetrics and Gynecology, Aristotle University of Thessaloniki, Tsimiski 51 Street, 54623 Thessaloniki, Greece

**Keywords:** Genital tract, Female genital anomalies, Mullerian anomalies, Uterine anomalies, ESHRE/ESGE system, Diagnosis, Classification

## Abstract

What is the recommended diagnostic work-up of female genital anomalies according to the European Society of Human Reproduction and Embryology (ESHRE)/European Society for Gynaecological Endoscopy (ESGE) system? The ESHRE/ESGE consensus for the diagnosis of female genital anomalies is presented. Accurate diagnosis of congenital anomalies still remains a clinical challenge due to the drawbacks of the previous classification systems and the non-systematic use of diagnostic methods with varying accuracy, with some of them quite inaccurate. Currently, a wide range of non-invasive diagnostic procedures are available, enriching the opportunity to accurately detect the anatomical status of the female genital tract, as well as a new objective and comprehensive classification system with well-described classes and sub-classes. The ESHRE/ESGE Congenital Uterine Anomalies (CONUTA) Working Group established an initiative with the goal of developing a consensus for the diagnosis of female genital anomalies. The CONUTA working group and imaging experts in the field have been appointed to run the project. The consensus is developed based on (1) *evaluation of the currently available diagnostic methods* and, more specifically, of *their characteristics* with the use of the experts panel consensus method and of *their diagnostic accuracy* performing a systematic review of evidence and (2) consensus for (a) the definition of where and how to measure uterine wall thickness and (b) the recommendations for the diagnostic work-up of female genital anomalies, based on the results of the previous evaluation procedure, with the use of the experts panel consensus method. Uterine wall thickness is defined as the distance between interostial line and external uterine profile at the midcoronal plane of the uterus; alternatively, if a coronal plane is not available, the mean anterior and posterior uterine wall thickness at the longitudinal plane could be used. Gynaecological examination and two-dimensional ultrasound (2D US) are recommended for the evaluation of asymptomatic women. Three-dimensional ultrasound (3D US) is recommended for the diagnosis of female genital anomalies in “symptomatic” patients belonging to high-risk groups for the presence of a female genital anomaly and in any asymptomatic woman suspected to have an anomaly from routine avaluation. Magnetic resonance imaging (MRI) and endoscopic evaluation are recommended for the sub-group of patients with suspected complex anomalies or in diagnostic dilemmas. Adolescents with symptoms suggestive for the presence of a female genital anomaly should be thoroughly evaluated with 2D US, 3D US, MRI and endoscopy. The various diagnostic methods should be used in a proper way and evaluated by experts to avoid mis-, over- and underdiagnosis. The role of a combined ultrasound examination and outpatient hysteroscopy should be prospectively evaluated. It is a challenge for further research, based on diagnosis, to objectively evaluate the clinical consequences related to various degrees of uterine deformity.

## Introduction

Female genital malformations are deviations from normal anatomy that could impair the reproductive potential of a woman or, in complex cases (e.g. obstructing anomalies), woman’s health [8, 12, 20, 21, 24, 25, 32, 43, 52, 56, 59]. They arise embryologically from failure of Müllerian ducts’ formation, canalization, fusion or absorption either as a single defect or in combination with different expression in the various parts of the female genital tract resulting in the so-called complex anomalies.

Accurate diagnosis of congenital anomalies still remains a clinical challenge with serious consequences in the management of those patients. This is the result of the following methodological bias: (1) absence of clear definitions and objective diagnostic criteria in the existing classification systems, mainly that of the American Fertility Society [1] for their diagnosis and differential diagnosis and (2) use of diagnostic methods with different accuracy, some of them quite inaccurate to make the correct diagnosis of the anomaly [54]. Thus, over the years, different investigators adopted their own subjective criteria, for the categorization of mainly uterine anomalies, that varied widely from one study to another, having as a result a poor selection and definition of the various patients’ populations [54, 26, 16].

In view of these diagnostic methological and clinical drawbacks, the estimation of their exact prevalence in the general and selected populations was very difficult and the evaluation of the clinical consequences of each different types of anomaly inaccurate [54, 11]. Furthermore, comparisons between different studies and their grouping are hampered not only by the differences in study populations but also by differences in diagnostic methods and criteria used to differentiate between various types of uterine anomalies [59]. Moreover, the exact value of surgery is not known for patients’ counselling and treatment underlying the urgent need to test available interventions in well-designed studies with properly defined groups [59].

In the recently published European Society of Human Reproduction and Embryology (ESHRE)/European Society for Gynaecological Endoscopy (ESGE) classification of female genital anomalies, a clear definition of all types of anomalies was provided and the anomalies were categorised in well-described classes and sub-classes [27, 28]. Thus, the previously mentioned diagnostic drawback of subjectivity in definitions is effectively answered enhancing their objective categorization [27, 28, 16]. It seems that with the use of the new system, all the existing, previously AFS poorly described and un-classified cases could be effectively described and classified with very rare exceptions offering a common “language” of communication between the clinicians working in this field [16].

Currently, a wide range of non-invasive diagnostic procedures are available enriching the opportunity to detect the anatomical status of the female genital tract in an accurate way. However, the various existing methods have different characteristics, availability, invasiveness and diagnostic accuracy [5, 11, 54]. Thus, it is important to clarify their current role in the diagnostic work-up and objective documentation of female genital tract anomalies. Furthermore, standardised and systematic evaluation of asymptomatic women and of selected “high-risk” populations for the presence of female genital anomalies is fundamental for their management.

The aim of the Thessaloniki ESHRE/ESGE consensus is to provide the researchers with recommendations for the diagnostic work-up of female genital anomalies; the definitions of the ESHRE/ESGE classification were used as basis for their development. This is an initiative of the Congenital Uterine Anomalies (CONUTA) Working Group, which was started during the ESHRE Campus Workshop on Female Genital Anomalies in Thessaloniki.

### Strategy for the consensus development

The development of the Thessaloniki ESHRE/ESGE consensus for the diagnosis of female genital anomalies by the CONUTA Working group was designed as follows:

1. *Evaluation of the currently available diagnostic methods*, including
  - *The evaluation of the characteristics* of each different currently available diagnostic technique by the group of invited imaging experts and the members of the CONUTA group with the use of the *experts panel consensus method* [33]; a draft was circulated in two rounds for comments and a live meeting was arranged for the consensus
  - *The evaluation of the diagnostic accuracy* of the different diagnostic methods performing a *systematic review of evidence* by SS, ADS and GG and
2. *Consensus development,* based on the results of the evaluation procedure, *including*
  - *the definition of where and how to measure uterine wall thickness* by the invited imaging experts and the members of the CONUTA group with the use of the *experts panel consensus method*; a draft was circulated in two rounds for comments and a live meeting was arranged for the consensus
  - *The recommendations* for the diagnostic work-up of female genital anomalies with the use of the *experts panel consensus method*, an initial proposal was circulated and the final document was prepared based on the comments.

The final document, including all the parts, was circulated again for final comments and approval from all the members of the consensus.

## Evaluation of the currently available diagnostic methods

### Diagnostic methods and their characteristics (consensus between experts)

#### Background

Anatomy of the female genital tract is the basis of the ESHRE/ESGE classification system. More specifically, *uterine anatomy* is the basis for the *main classes and sub-classes*. Cervical and vaginal anomalies are classified independently in supplementary subcategories. Thus, diagnosis of uterine anomalies has to be based on diagnostic modalities that determine the anatomical status of the female genital tract in an objective way.

Each diagnostic method should ideally provide objective and measurable information on the anatomical status of: (i) the vagina, (ii) the cervix, (iii) the uterine cavity, (iv) the uterine wall, (v) the external contour of the uterus and (vi) the other intra-peritoneal structures.

#### Question

What is the diagnostic potential, advantages, disadvantages and way of proper use of the available imaging techniques in the diagnosis of female genital tract congenital anomalies?

### Gynaecological examination

#### Diagnostic potential inherent to the method

*Some vaginal and some cervical* malformations (aplasia, double cervices, longitudinal septa reaching to the external cervical os) can be diagnosed *objectively by inspection. Palpation* (through the vagina and/or the rectum in cases of vaginal aplasia) *cannot* provide *information* for the *uterine cavity and uterine wall* and could provide only some useful, but *highly subjective, information* for the uterine body (e.g. complete bicorporeal uterus). *Palpation* could provide information *in cases of dilatation secondary to obstruction of menstrual flow* (hematocolpos/hematometra/hemato-cavity in cases of non-communicating uterine horns).

#### Advantages

Gynaecological examination is always *the starting point and an essential part* of any woman’s clinical evaluation. It is non-invasive, simple, easy and low cost. It offers *unique information* in cases of some vaginal and cervical anomalies; it is also crucial that vaginal examination could elicit tenderness, which can aid diagnosis. It is included in the basic training of Obstetricians and Gynaecologists needing *no additional expertise*.

#### Disadvantages

It should not be used for the diagnosis of uterine anomalies due to its inherent inability to provide reliable information for uterine anatomy. It is not a primary approach in women who have never been sexually active.

#### Recommendations for its proper use

In cases of primary amenorrhea, careful inspection of the external genitalia for the presence of distal vaginal aplasia. Careful inspection of the vagina, to avoid mis-diagnosis in cases of longitudinal vaginal septa, by entering only in one of the two existing vaginal spaces. Careful inspection of the vaginal vault with a speculum to establish the presence of one or more cervical body(ies) or one cervical body with one or two external cervical opening(s). In cases of cyclic pelvic pain, with or without primary amenorrhea, careful palpation for palpable masses secondary to accumulation of menstrual blood (obstructed parts).

### X-ray hysterosalpingography

#### Diagnostic potential inherent to the method

It *provides some reliable information* for the anatomy of the *uterine cavity* in the absence of cervical obstruction. It *could provide, also, information* for the anatomy of the *cervical canal* in the absence of cervical obstruction; the information on the anatomy of the cervical canal may be limited due to the instruments placed within and in the vicinity of the cervix. It *does not provide* any information for *the vagina* (exception: blind vagina with small opening)*, the uterine wall and the external contour of the uterus*. It *does not provide any information* for rudimentary *non-communicating horns or cavities.*

#### Advantages

It is widely available and offers printable films that could be re-evaluated anytime. It offers additional useful information in cases of infertile women for potential intra-cavitary pathology (presence of defects/differential diagnosis between adhesions, polyps, myomas) and tubal morphology

#### Disadvantages

Its disadvantages include painful, risk of infection and irradiation of the patient. It is *more invasive than ultrasound*, not always easy and *needing radiological unit*. It *cannot be used for the differential diagnosis of uterine anomalies* due to its inherent inability to provide reliable information for uterine wall and the uterine outline anatomy; uterine anomalies represent the vast majority of malformations. Its diagnostic accuracy is restricted by false-positive and false-negative results; air bubbles might be mistaken for intra-cavity pathology; distension of the cavity due to fluid injection might distort the shape of the cavity to a degree that is related to whether there is a tubal ostia obstruction or not and, hence, limiting the value of assessing the interior contour. It *cannot be used for the diagnosis of obstructing anomalies.*

#### Recommendations for its proper use

The examiner has to be very cautious in order to be precise: *pulling the uterus is necessary* for the best imaging of the uterine cavity (otherwise small indentations could be missed). *Careful inspection of the vagina and the cervix* must be done to avoid mis-diagnosis in cases of double or septate cervix with or without longitudinal vaginal septa; *catheterization of both cervical* canals, if present, is necessary.

### Two-dimensional ultrasound

#### Diagnostic potential inherent to the method

It could provide *reliable, objective and, most importantly, measurable information* for the anatomy of the *cervix, uterine cavity, uterine wall and external contour of the uterus.* It could provide *useful information of associated pelvic pathology*, e.g. ovarian pathology (e.g. benign and malignant tumours, endometriosis), hydrosalpinges, renal anomalies etc. It could provide, also, measurable information even for obstructing parts of the female genital tract. Transperineal 2D ultrasound may provide information on the vaginal cavity, especially in the presence of imperforate hemivagina.

#### Advantages

It is non-invasive, simple, low cost and available in almost every setting. *Gynaecologists are familiar* with the technique since training in ultrasound is included in the basic training in obstetrics and gynaecology; nowadays, *ultrasound* examination is an essential *part of women’s routine evaluation*. Electronic storage of the diagnostic procedure is nowadays feasible for re-evaluation. It could provide the required planes in a flexible way since the examiner could change the position of ultrasound probes according to the needs of imaging. It offers additional valuable information in cases of infertile women for potential intra-cavitary (major adhesions might be supsected presented as “bridges” between the walls, polyps, myomas) and intramural pathology (myomas, adenomyosis).

#### Disadvantages

The *diagnostic accuracy of two-dimensional ultrasound (2D US)* being a dynamic examination, is highly dependent on the *experience of the examiner* and on the proper and *systematic way* of performing the procedure. It is not always feasible to have the required planes due to the patient’s anatomical characteristics.

#### Recommendations for its proper use

The endometrial line should be well visible for precise imaging of the uterine cavity (late proliferative or secretory phase or intra-cavitary fluid enhancement/avoid early follicular phase). *Serial sagittal planes* from beyond the outer margin of one side of the uterus to the other including both cervix and uterine body if feasible *and transverse planes* from the cervix to beyond the uterine fundal level should be taken in a systematic way. In cases of vaginal obstruction or stenosis, if the woman consents, *transrectal ultrasound with vaginal probe or transperineal* could be performed to evaluate vaginal canal and uterus (not in children nor in adolescents). Abdominal palpation should be applied to improve the image by pushing away the bowel and to assess mobility of the pelvic organs; gynaecologists are better able to do this compared with sonographers.

### Hysterosalpingo-contrast sonography

#### Diagnostic potential inherent to the method

It can provide *reliable, objective and, most importantly, measurable information* for the anatomy of the *cervix, uterine cavity, uterine wall, external contour of the uterus and* for other peritoneal structures (e.g. ovaries) with the exception of tubes. The *imaging of uterine cavity is better* due to the use of the contrast medium or saline enhancing the accuracy in identifying uterine cavity defects. Hysterosalpingo-contrast sonography could be used as a *tubal patency test* (infertile patients).

#### Advantages

It is minimally invasive, simple, low cost, potentially available in almost every setting (since only contrast medium is needed). *Gynaecologists could easily apply* the technique since training in ultrasound is included in the basic training in obstetrics and gynaecology, and insertion of an intra-uterine catheter could be done easily by them. *Electronic storage* of the diagnostic procedure is, nowadays, feasible for re-evaluation. It could provide *the required planes in a flexible way* since the examiner could change the position of ultrasound probes according to the needs of imaging. It offers *additional, more reliable information than that of 2D US* in cases of infertile women for *potential intra-cavitary* (adhesions presented as “bridges” between the walls, polyps, myomas) and *intramural pathology* (myomas, adenomyosis) but not necessarily for uterine malformations.

#### Disadvantages

The *diagnostic accuracy of hysterosalpingo-contrast sonography (HyCoSy)*, being a dynamic examination, is highly dependent on the *experience of the examiner* and on the proper and *systematic way* of performing. Distension of the uterine cavity could potentially modify internal uterine contour resulting *in false-negative imaging of the uterine cavity* especially in marginal uterine anomalies. It is not always feasible to have the required planes due to the patient’s anatomical characteristics. It is rarely painful with difficulties in the insertion of the catheter.

#### Recommendations for its proper use

Early follicular phase is recommended as appropriate to avoid pregnancies and artefacts due to thick secretory endometrium. *Serial sagittal planes* from beyond the outer margin of one side of the uterus to the other including both cervix and uterine body if feasible *and transverse planes* from the cervix to beyond the uterine fundal level should be taken in a systematic way

### Three-dimensional ultrasound

#### Diagnostic potential inherent to the method

It can provide *highly reliable, objective and, most importantly, measurable information* for the anatomy of the *cervix, uterine cavity, uterine wall, external contour of the uterus* and for associated pelvic pathology; the coronal plane of the uterus does provide a clear image of the cavity and *the external profile of the uterine fundus*. 3D volumes give *reliable and objective representation* of the *examined* organs *more independently of the examiner* overcoming the limitations of obtaining coronal images with 2D sonography. It can provide, also, measurable *information even for obstructed parts* of the female genital tract.

#### Advantages

It is non-invasive and easily applied to the patient (no difference from conventional ultrasound). *Reliable imaging of the uterus* since *uterine anatomy is presented* in the sagittal, transverse and coronal planes in an objective way independently of the examiner’s ability. It provides *precise and objective measurements of the uterine dimensions* which is the absolute advantage in differential diagnosis between different classes. *Electronic storage* of the volume is, nowadays, routinely done for re-evaluation giving the opportunity for off-line analysis enabling the assessment of the uterus/uterine wall in different slices and to choose the plane of maximum interest in the coronal/sagittal or transverse sections for measurements. It offers *additional information, which is more reliable than that of 2D US,* in cases of infertile women for potential intra-cavitary (adhesions presented as “bridges” between the walls, polyps, myomas) and intramural pathology (myomas, adenomyosis). *Transperineal three-dimensional ultrasound (3D US)* may offer the opportunity to view pelvic structures including the vagina and cervix.

#### Disadvantages

It is not so widely available as 2D US (up to now). It needs experienced sonographers with *special and adequate training* in 3-dimensional image acquisition and post-processing techniques. *Bewarefor artefacts* due to inappropriate volume acquisition and/or manipulation of the volume. It cannot provide very detailed and reliable data in very few cases of complex anomalies. 3D US without saline infusion or contrast medium cannot be used as a real time tubal patency test in cases of infertile patients.

#### Recommendations for its proper use

This method should be started with a 2D evaluation of the uterus. Use in midcycle or luteal phase is encouraged as this demonstrates the endometrial wall and the outline of the cavity at its best. Contrast medium could be used for the evaluation of the cavity and the tubes; in these cases, the examination has to be performed in the early follicular phase. Save a 3D volume for off-line analysis. The reconstructed coronal plane of the uterus might show the cavity and the external uterine profile as well as the tubal angle and the junctional zone, if possible along all the endometrium and cavity. Acquisition of an isolated cervical volume, without including the uterus: from a mid-sagittal plane, an axial plane of cervix can be obtained in 80 % and a coronal plane in 20 % of the cases; in cases of uterine malformations, the extent of the cervix and the limits of the cervical canal may be studied better. Diagnosis of associated vaginal anomalies can be done by transperineal acquisition of the pelvic floor volume after filling the vagina with gel or saline; an axial plane can be obtained from a mid-sagittal plane.

### Magnetic resonance imaging

#### Diagnostic potential inherent to the method

It can provide *highly reliable and objective information* for the anatomical status of the vagina *cervix, uterine cavity, uterine wall, external contour of the uterus* and for other peritoneal structures with the exception of tubes. It provides, also, *reliable information even for dilated (obstructed) parts* of the female genital tract.

#### Advantages

It is non-invasive and it has no radiation. It gives *a reliable and objective representation* of the examining organs *in the sagittal, transverse and coronal plane* (three dimensions)*.* It can be used for *diagnosis in cases of complex and obstructing anomalies. Electronic storage* of the diagnostic procedure is, nowadays, routinely done for re-evaluation

#### Disadvantages

It is more expensive and less available than ultrasound and not appropriate for patients with claustrophobia and morbid obesity. It needs *experience and training* in the assessment of the results. *The required planes are provided in a non-flexible way* since planes are pre-defined and independent of the examiner, a disadvantage that could potentially impair the diagnostic accuracy of the method in the absence of an experienced radiologist. It cannot be used as a tubal patency test in cases of infertile patients.

#### Recommendations for its proper use

Gynaecologists should be trained in magnetic resonance imaging (MRI) reading and work closely with radiologists to review the images as the clinical background knowledge of the former supplements the radiological interpretation of the images by the latter.

### Hysteroscopy

#### Diagnostic potential inherent to the method

It provides *highly reliable information* for the anatomical status of the vagina (vaginoscopic approach), the *cervical canal and, mainly, the uterine cavity and the tubal ostia.*

#### Advantages

It is minimally invasive giving the additional *opportunity of treating T-shaped, septate and bicorporeal septate uterus. Its objective includes estimation of the cervical canal and endometrial cavity* (differential diagnosis of T-shaped and infantile uterus). It provides a minimal invasive *evaluation of the vagina and/or cervix in case of virgo. Electronic storage* of the procedure is, nowadays, routinely done for re-evaluation.

#### Disadvantages

It is more complex to organise but includes *no information for uterine wall thickness and uterine outline* and is unable to offer differential diagnosis between septate and bicorporeal uterus. It needs *experience and training*. Evaluation of the cavity is not feasible in cases of obstructed anomalies. It could not be used as a tubal patency test in cases of infertile patients.

#### Recommendations for its proper use

It complements ultrasound in the initial investigation of female genital tract malformations.

### Endoscopy; laparoscopy and hysteroscopy

#### Diagnostic potential inherent to the method

It provides *highly reliable information* for the anatomical status of the vagina (vaginoscopic approach), *cervical canal, uterine cavity, tubal ostia, external contour of the uterus and the intra-peritoneal structures.*

#### Advantages

*It is a direct visualisation of the cervical canal, endometrial cavity* and the *external contour* of the uterus representing until now the *“gold standard”* in the diagnosis and differential diagnosis. *Electronic storage* of the procedure is, nowadays, routinely done for re-evaluation. Endoscopic approach represents the minimally invasive route of choice in the *treatment of a wide variety of female genital anomalies.*

#### Disadvantages

It is invasive with no objective estimation of the uterine wall thickness. The diagnosis is mainly based on the subjective impression of the clinician performing them, and this is thought to be a limitation in the objective estimation of the anomaly. It needs *experience and training*.

#### Recommendations for its proper use

The invasiveness of the laparoscopic approach makes it not acceptable as a first-line screening procedure; it complements indirect imaging in the diagnosis of more complex anomalies in combination with possible surgical actions. It offers supplementary information about partial or total absence of Fallopian tubes and abnormal localization of ovaries.

### Computerized tomography scanning (CTS)

Computerized tomography scanning (CTS) has no place any longer in the diagnosis of female genital anomalies due to radiation and poor depiction of the female genital structures and it was not included in the evaluation.

### Diagnostic accuracy of the different methods (systematic review of evidence)

#### Question

What is the diagnostic accuracy of the available imaging techniques in the diagnosis of female genital tract congenital anomalies as compared to the combined hysteroscopic and laparoscopic investigation (reference standard) based?

#### Limitations

Prior to approaching this problem, the limitations have to be recognised and disclosed as follows. Firstly, the studies to date will not have based the assessment of different diagnostic accuracies on the current ESHRE/ESGE classification. Therefore, evidence will inevitably have to be drawn from the period following the initial Buttram and Gibbons classification [9], which was later revised into the American Fertility Society classification [1], the most widely accepted classification worldwide for the last 25 years.

Secondly, the gold standard method of comparison for diagnosis to date has been the combined hysteroscopy and laparoscopy investigation, which allows for the direct visualisation of the internal and external contour of the uterus but does not always allow accurate and objective uterine measurements. With the new ESHRE/ESGE classification and need to measure fundal, septal and lateral uterine wall thickness, it might be possible and necessary that the gold standard test may evolve to become another imaging modality in the future.

#### Methods

**Fig. 1:**
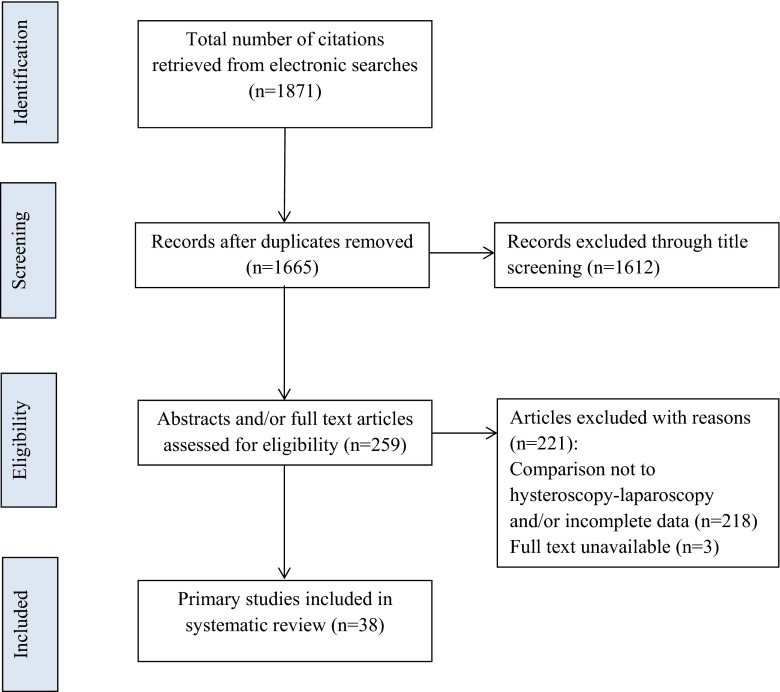
The study selection process for the systematic review on the diagnostic accuracy of the different methods used to assess female genital anomalies

Articles assessing the diagnostic accuracy of the most widely used imaging techniques were searched through MEDLINE, EMBASE and the Cochrane Library from 1988 to 2014. A combination of text words and Medical Subject Headings (MeSH) were used to generate the list of citations (Table 1); these were primarily designed for MEDLINE and were modified appropriately for EMBASE and the Cochrane Library. In addition to the electronic searches, relevant articles were hand searched from further citations. The study selection process is shown in Fig. 1.

**Table 1:** Search terms used in the systematic review (either as MeSH terms or free text terms)

Uterus/abnormalities (MeSH)	Ultrasonography (MeSH)
Mullerian ducts/abnormalities (MeSH)	Hysterosalpingography (MeSH)
Female genital abnormalit^a^	Magnetic resonance imaging (MeSH)
Female genital anomal^a^	Hysteroscopy (MeSH)
	Laparoscopy (MeSH)

^a^Any character

The diagnostic accuracy was estimated by combining the values of sensitivity, specificity, positive predictive value (PPV) and negative predictive value (NPV) of each imaging technique according to the formula of Altman [14]; as reference standard was used the combined hysteroscopic and laparoscopic investigation. When studies did not report these values in text, 2 × 2 tables were manually constructed where possible, and these variables were individually estimated. Data were analysed on IBM SPSS version 21 for Windows (SPSS Inc., IL, USA). Means and 95 % confidence intervals (CI) for sensitivity, specificity, PPV and NPV and accuracy were calculated for each individual methodology.

Primary outcome of this systematic review was the accuracy of each diagnostic method in terms of identifying a congenital malformation.

#### Results

Thirty-eight studies of high quality were included in the primary analysis. Several studies were excluded due to inadequate gold standard methodology used and incomplete/absent data regarding the diagnostic accuracy. There were no studies found reporting on the use of MRI as a screening tool (studies included patients with a previous diagnosis of congenital malformations undergoing further evaluation), and therefore the secondary outcome but not the primary outcome could be assessed for this methodology.

Pooled analysis of the included studies showed that the highest degrees of overall diagnostic accuracy were in decreasing order: 3D US (97.6 %), sonohysterography (SHG; 96.5 %), 2D US (86.6) and hysterosalpingography (HSG; 86.9 %). MRI was shown to be able to correctly subclassifiy 85.8 % of anomalies, which implies that the accuracy of identifying the presence of a malformation is well above 90 % (Tables 2, 3, 4, 5 and 6). Overall, it appears that 3D US may be more accurate than MRI in sub-classifying malformations, although it should be noted that sub-classification is hindered due to the subjective nature of the previous classifications adopted.

**Table 2:** Diagnostic accuracy of HSG compared with hysteroscopy ± laparoscopy in diagnosing female genital tract congenital anomalies

Study	Cases (*n*)	Sensitivity	Specificity	PPV	NPV	Accuracy
Bocca et al. [6]	125	50	94	71	87	76
Ludwin et al. [37]	83	77	100	100	35	78
De Felice et al. [14]	208	100	100	100	100	100
Momtaz et al. [44]	38	95	78	65	97	84
Guimaraes Filho et al. [30]	54	63	98	83	94	85
Valenzano et al. [58]	54	91	100	100	94	96
Traina et al. [57]	80	100	97	85	100	96
Alborzi et al. [3]	186	70	92	83	88	83
Preutthipan and Linasmita [48]	336	100	97	69	100	92
Brown et al. [7]	46	100	100	100	100	100
Soares et al. [55]	65	44	96	67	92	75
Alatas et al. [2]	62	100	100	100	100	100
Garglione 1997	70	100	100	100	100	100
Goldberg et al. [23]	32	100	100	100	100	100
Keltz et al. [34]	18	90	20	53	67	58
Raziel et al. [51]	60	74	59	62	72	67
Mean (95 % CI)		84.6 (74.4–94.9)	89.4 (80.0–100)	83.6 (74.6–92.6)	89.1 (79.7–98.5)	86.9 (79.8–94.0)

*HSG* hysterosalpingogram, *PPV* positive predictive value, *NPV* negative predictive value, *CI* confidence interval

**Table 3:** Diagnostic accuracy of 2D US compared with hysteroscopy ± laparoscopy in diagnosing female genital tract congenital anomalies

Study	Cases (*n*)	Sensitivity (%)	Specificity (%)	PPV (%)	NPV (%)	Accuracy (%)
Ludwin et al. [38]	117	91	92	99	52	84
De Felice et al. [14]	104	100	99	86	100	96
Momtaz et al. [44]	38	55	95	84	83	79
Valenzano et al. [58]	54	86	100	100	91	94
Ragni et al. [50]	98	73	100	100	97	93
Traina et al. [57]	80	64	99	88	94	86
Soares et al. [55]	65	44	100	100	92	84
Alatas et al. [2]	62	50	100	100	97	87
Nicolini et al. [46]	89	43	98	94	68	76
Mean (95 % CI)		67.3 (51.0–83.7)	98.1 (96.0–100)	94.6 (89.4–99.8)	86.0 (73.7–98.3)	86.6 (81.3–91.8)

*2D US* two-dimensional ultrasound, *PPV* positive predictive value, *NPV* negative predictive value, *CI* confidence interval

**Table 4:** Diagnostic accuracy of HyCoSy compared with hysteroscopy ± laparoscopy in diagnosing female genital tract congenital anomalies

Study	Cases (*n*)	Sensitivity (%)	Specificity (%)	PPV (%)	NPV (%)	Accuracy (%)
Ludwin et al. [38]	117	94	83	99	65	85
Ludwin et al. [37]	83	96	89	99	73	89
De Felice et al. [14]	104	100	100	100	100	100
Guimaraes Filho et al. [30]	55	100	94	73	100	92
Valenzano et al. [58]	54	100	100	100	100	100
Ragni et al. [50]	98	91	100	100	99	98
Alborzi et al. [3]	186	91	100	100	96	97
Dodero et al. [17]	52	100	100	100	100	100
Brown et al. [7]	46	100	100	100	100	100
Soares et al. [55]	65	73	100	100	97	93
Alatas et al. [2]	62	100	100	100	100	100
Goldberg et al. [23]	32	100	100	100	100	100
Keltz et al. [34]	18	100	100	100	100	100
Mean (95 % CI)		95.8 (91.1–100)	97.4 (94.1–100)	97.8 (93.3–100)	94.6 (87.6–100)	96.5 (93.4–99.5)

*HyCoSy* hysterosalpingo-contrast sonography, *PPV* positive predictive value, *NPV* negative predictive value, *CI* confidence interval

**Table 5:** Diagnostic accuracy of 3D US compared with hysteroscopy ± laparoscopy in diagnosing female genital tract congenital anomalies

Study	Cases (*n*)	Sensitivity (%)	Specificity (%)	PPV (%)	NPV (%)	Accuracy (%)
Imboden et al. [31]	10	100	100	100	100	100
Laganà et al. [35]	224	100	100	100	100	100
Ludwin et al. [38]	117	97	100	100	80	94
Moini et al. [42]	214	87	97	99	54	84
Bocca et al. [6]^a^	125	100	100	100	100	100
Faivre et al. [18]	31	100	100	100	100	100
Ghi et al. [22]	284	100	100	100	100	100
Makris et al. [39]	248	100	100	100	100	100
Momtaz et al. [44]	38	97	96	92	99	96
Radoncic and Funduk-Kurjak [49]	267	100	100	100	100	100
Wu et al. [61]	40	100	100	100	100	100
Mean (95 % CI)		98.3 (95.6–100)	99.4 (98.4–100)	99.2 (97.6–100)	93.9 (84.2–100)	97.6 (94.3–100)

*3D US* three-dimensional ultrasound, *PPV* positive predictive value, *NPV* negative predictive value, *CI* confidence interval

^a^Performed in conjunction with saline infusion

**Table 6:** Diagnostic accuracy of MRI compared with hysteroscopy ± laparoscopy in diagnosing female genital tract congenital anomalies

Study	Cases (*n*)	Correct sub-classification (*n*; %)
Imboden et al. [31]	13	7/13 (54 %)
Faivre et al. [18]	31	24/31 (77 %)
Santos et al. [53]	26	23/26 (89 %)
Mueller et al. [45]	105	83/105 (81 %)
Deutch et al. [15]	7	2/7 (29 %)
Marten et al. [40]	4	4/4 (100 %)
Console et al. [13]	22	21/22 (95 %)
Minto et al. [41]	9	7/9 (78 %)
Letterie et al. [36]	16	12/16 (75 %)
Pellerito et al. [47]	24	24/24 (100 %)
Carrington et al. [10]	29	29/29 (100 %)
Fedele et al. [19]	18	18/18 (100 %)
Weighted mean		254/296 (85.8 %)

*MRI* magnetic resonance imaging;

Sensitivity, specificity, PPV and NPV cannot be assessed for MRI as this was not used as a *screening* tool in the studies identified

## Consensus development

### Measurement of the uterine wall thickness (consensus between experts)

#### Background

Uterine wall thickness *is an important parameter and a reference point* for the definitions of dysmorphic T-shaped, septate and bicorporeal uteri according to the new classification system. The adoption of an objective criterion for the definition of uterine deformity is one of the advantages of the new classification system since according to AFS classification the detection of anomalies was based only on the subjective impression of the clinician performing the test. Although myometrial thickness at the various uterine regions cannot be easily assessed with endoscopic techniques, *it can be measured* with *ultrasound or MRI*.

However, the thickness of the uterine wall as the reference value for the estimation of the internal indentation at the mid-fundal level in cases of septate uterus, external indentation in cases of bicorporeal and lateral wall thickness in cases of T-shaped uterus might, indeed, vary in different regions of the uterus. Thus, recommendations for the measurement of uterine dimensions and accurate description of uterine deformity are very important.

#### Question

Where and how to measure the reference value of the uterine wall thickness?

#### Main option

This include the distance between the interostial line and external uterine profile at the mid-coronal plane of the uterus (fitted to 3D US, MRI and, at times, 2D US).

#### Definition of the reference value of the uterine wall thickness

This is the distance between the line connecting the tubal ostia and the external uterine profile *obtained with 3D US, MRI and, at times, with 2D US. Comments:* in cases of an external indentation (fusion defects), the distance between the two lines: one connecting the tubal ostia and the other the external outline of the two uterine bodies.

#### Why to use this as a reference parameter

Uterine anomalies are (fusion and/or absorption) defects at the uterine fundal midline and, therefore, measurements should be oriented there. Until now, imaging at that level has always been used until now to diagnose congenital uterine anomalies.

#### How to measure (Figs. 2, 3a-c and 4a-d):

**Fig. 2:**
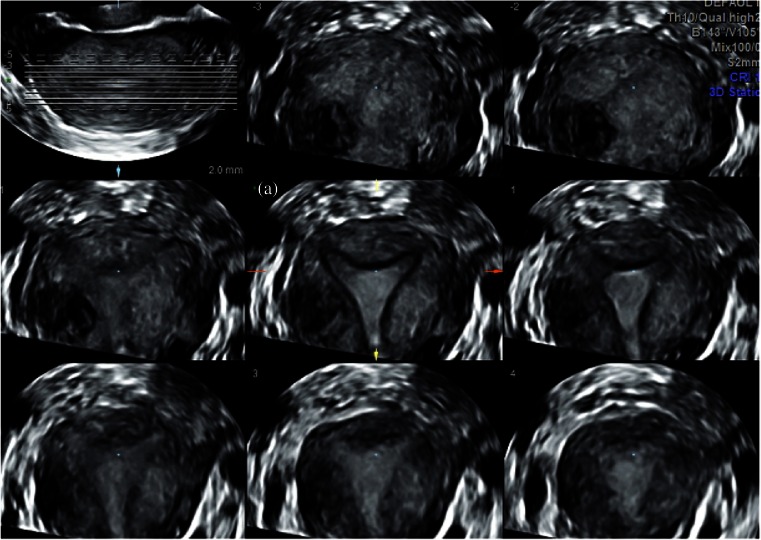
How to obtain an optimal 3D US coronal plane: tomographic ultrasound imaging (TUI) is the representation by a series of parallel slices through the volume and the distance between the slices as well as their number can be configured; the plane is optimal only if the slices or cutting line is exactly on the endometrium and the junctional zone at the level of the tubal ostia and isthmus (a) at the central plane

**Fig. 3:**
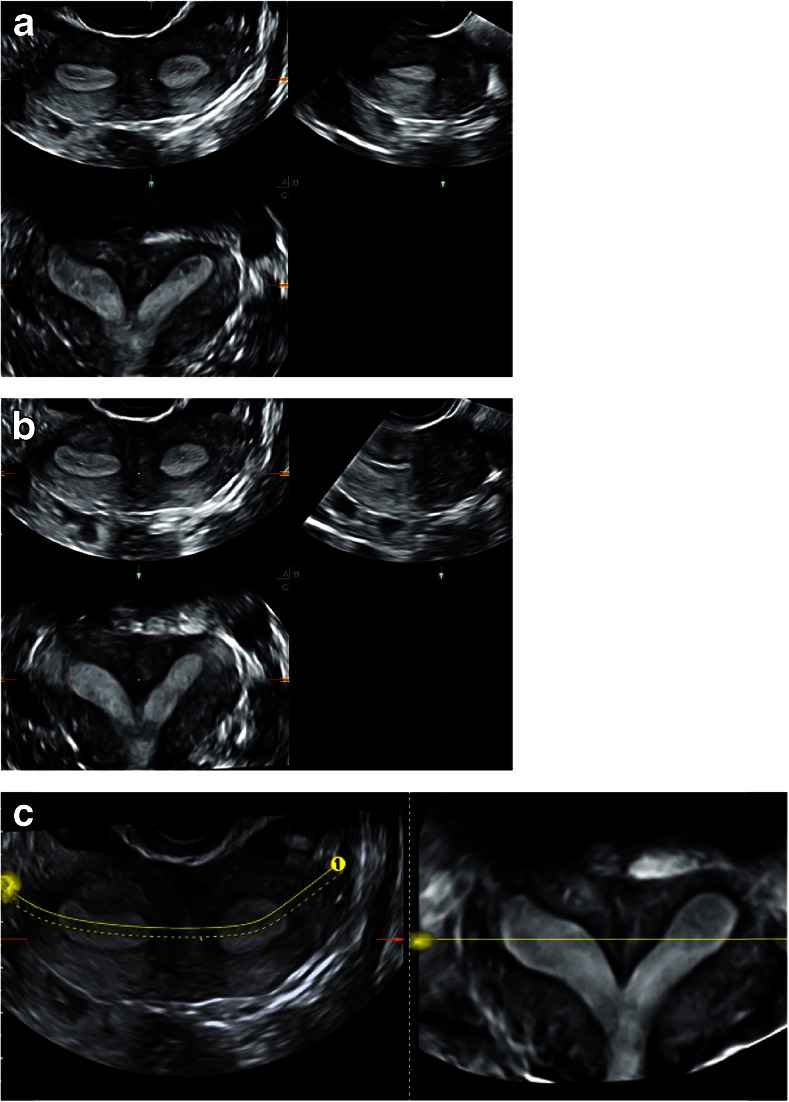
**a**–**c** How to obtain an optimal 3D US coronal plane: cutting line is not perfect on the endometrium in (**a**) and (**b**); thus, if necessary, the *dotted line* can be curved to follow the endometrium and the tubal ostia like in plane (**c**)

**Fig. 4:**
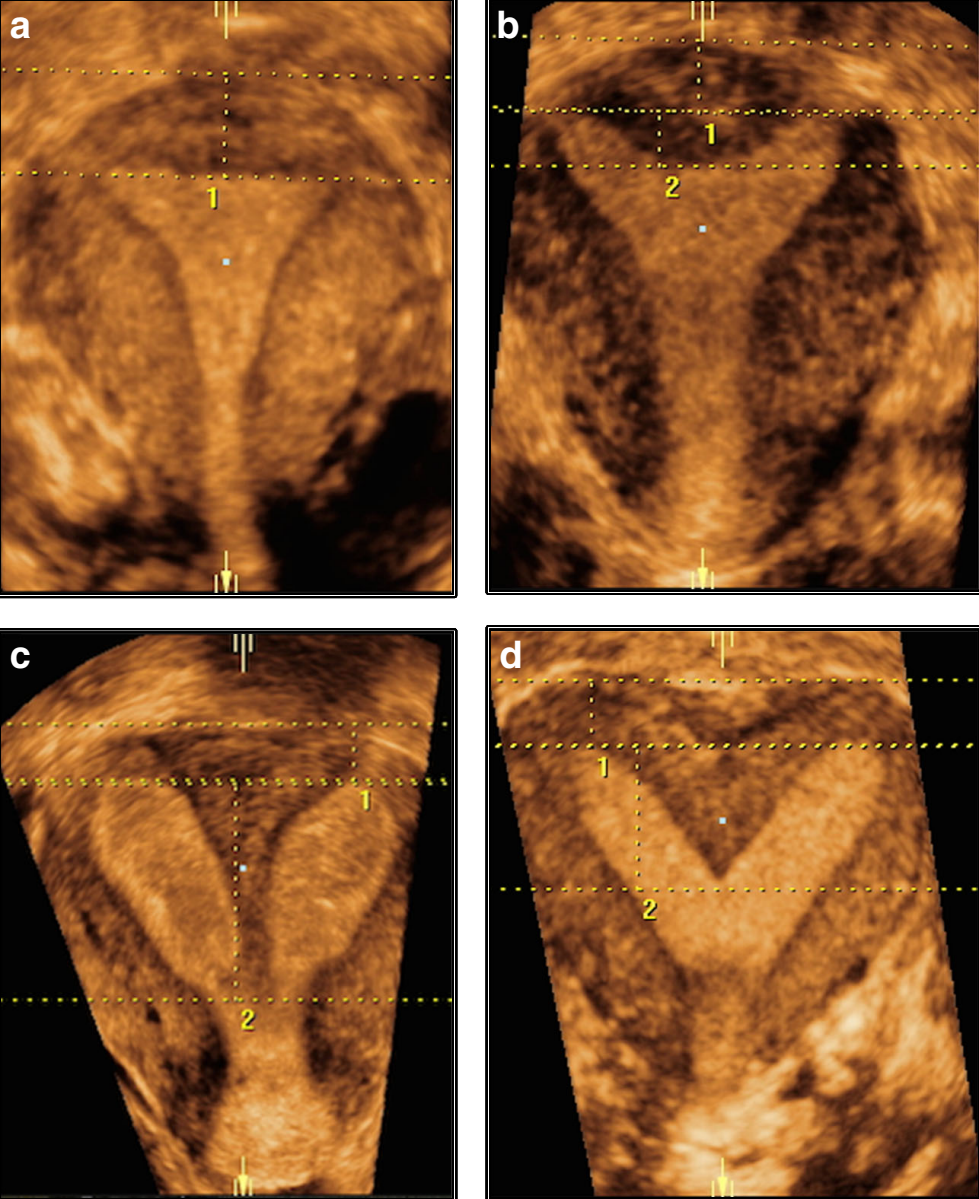
(**a**) Coronal 3D US view of a normal uterus; uterine wall thickness: *distance between the line joining tubal ostia (interostial line) and a parallel line on the top of the fundus*. (**b**) Coronal 3D US view of a partial septate uterus; *1*, uterine wall thickness: distance between the line joining tubal ostia (interostial line) and a parallel line on the top of uterine fundus; *2*, internal midline indentation: distance between the interostial line and a parallel line on the top of midline indentation. (**c**) Coronal 3D US view of a complete septate uterus: *1*, uterine wall thickness: distance between the line joining tubal ostia (interostial line) and a parallel line on the top of uterine fundus; *2*, internal midline indentation: distance between the interostial line and a parallel line on the top of midline indentation (the line reaches the internal cervical os). (**d**) Coronal 3D US view of a bicorporeal septate uterus: uterine wall thickness: distance between the interostial line and a parallel line joining the external outline of the uterine horns

Step 1 Imaging of the uterus in a midcoronal plane; a sectional plane or a rendered 3D ultrasound image of a coronal section of the uterus is now widely accepted as the most accurate plane for measurements.
Step 2 Draw the line connecting the two tubal ostia; in cases of an external indentation, draw a second line connecting the external profile of the two uterine bodies.
Step 3 In cases of patients with normal external uterine surface, the distance between the line connecting the tubal ostia and the external uterine outline is defined as the uterine wall thickness (reference value); in cases of patients with an existing external indentation, the distance between the two previously described lines is defined as the uterine wall thickness (reference value).
Step 4 Estimate the length of any existing internal indentation by measuring the distance between the interostial line and the indentation’s edge at the cavity; septum is considered any indentation >50 % of the previously measured total fundal uterine wall thickness. Estimate of the lateral wall thickness by measuring at an angle of 90° to the lining of the myometrial-endometrial border.

*Comments:* 1. Tubal ostia should be considered as the ultrasound border between uterine cavity and the proximal intramural part of the tubes. 2. *External uterine contour should be delineated clearly* in ultrasound images to avoid under- or overestimation of the uterine wall thickness. A non-rendered image in the C plane may give a sharper outline compared with a (thin) sliced rendered image.

##### *Drawbacks*

Drawback include the following: (1) When an anomaly is present, measurements in certain parts (fundus) could not be, sometimes, either feasible or representative, (2) external profile of the uterus at the fundal level is not always clearly assessable leading to an inaccurate evaluation and (3) in cases of bicorporeal uterus, sometimes the two uterine bodies are not very close to each other and this could create some diagnostic bias.

##### Alternative option

This include the mean thickness of the anterior and posterior uterine wall (fitted to 2D US).

#### Definition of the reference value of the uterine wall thickness

This is the mean thickness of the anterior and posterior wall in 2D or 3D US longitudinal planes at the mid-point of the uterine corpus. *Comments:* in cases of septate or bicorporeal uteri with an internal indentation covering more than 50 % of the uterine cavity, the longitudinal plane at the mid-cavity level is affected by the indentation and it could not be used as a reference plane for measurements. In that case, a longitudinal plane of the lateral cavities could be used as the reference for measurements in the same described way.

#### Why to use this as a reference parameter:

This part of the uterine wall could be considered as representative for measurements since it is not affected in cases of uterine anomalies and if it is affected alternatives could be provided.

#### How to measure:

Step 1 Imaging of the uterus in longitudinal plane,
Step 2 Estimation of mid-point between the fundal part of uterine cavity and the internal cervical os and
Step 3 Measurements of uterine wall thickness of the anterior and posterior wall at the mid-point level (estimated in step 2) taking the mean of those measurements as the reference point

#### Drawbacks

Drawbacks include the following: (1) Uterine wall thickness at the posterior, anterior and lateral uterine walls’ level is, probably, different from that observed at the fundal level even in the absence of any pathology, (2) uterine wall thickness at that level (mean of the anterior and posterior walls’ thickness on a longitudinal section) has never been used to define congenital uterine anomalies, (3) uterine wall thickness at the posterior and anterior level will be affected by a number of uterine conditions like fibromas and adenomyosis. Furthermore, with the vascular network placed laterally, the wall thickness might well be different and (4) uterine anomalies are (fusion and/or absorption) defects at the uterine fundal midline and, therefore, measurements should be oriented there.

### Recommendations (consensus between CONUTA group members and invited experts)

#### Background

Female genital anomalies are common benign entities with an estimating prevalence ranging from ∼6 % in the general population up to ∼15 % in selected population with recurrent pregnancy losses. Thus, women of reproductive age during their routine examination should be examined for the presence of a potential congenital anomaly. Certainly in symptomatic patients or, otherwise, in patients with higher risk for the presence of an anomaly, special attention should be paid during their diagnostic work-up.

The recommendations for the diagnostic work-up were based on the diagnostic potential of the different methods and their diagnostic accuracy. Additional parameters (e.g. accessibility, need for training and expertise, cost etc.) were also taken into account. The diagnostic methods should be used in a systematic way taking into consideration the comments for their proper use. The anatomical characteristics should be recorded and documented as described previously based on the anatomical varieties of the ESHRE/ESGE classification system.

#### Definitions

Asymptomatic patients: Patients consulting for routine gynaecological examination without complaints of chronic pelvic pain (i.e. dysmenorrhea, dyspareunia, cyclic low abdominal pain) and history of poor reproductive outcome having normal gynaecological findings at clinical examination.
Symptomatic or high risk patients: Groups of patients presenting with clinical problems that could be associated with the presence of female genital anomalies and expected to have higher prevalence than that of the general population. Thus, as symptomatic groups should be considered: (1) patients with *primary amenorrhea, inability of normal intercourse, chronic pelvic pain* (dysmenorhea, dyspareunia, cyclic abdominal pain); (2) patients *with poor reproductive outcome*, including (a) patients with *two or more IVF failures*, (b) women with *two or more 1st trimester pregnancy losses* and/or one *2nd trimester loss* and (c) women with a history of *preterm delivery*; and (3) *adolescents with symptoms* suggestive for the presence of a female genital anomaly.

#### Recommended evaluation of asymptomatic women

Clinicians should, always, be attentive for the presence of a congenital anomaly in asymptomatic women of reproductive age during their routine examination, supplementing gynaecological examination with a 2D US as follows:

- Gynaecological examination: the anatomy of the external genitalia, the vagina and the cervix should be carefully evaluated.
- 2D US: it should be done in a pre-defined and systematic manner to increase its diagnostic accuracy. The shape and the dimensions of the uterine cavity, the uterine wall (anterior, posterior, lateral and fundal width) and external uterine contour should be recorded in a systematic way in longitudinal and transverse planes.
- The absence of findings suspicious for the presence of an anomaly should not be considered as definite and the presence of one could not be excluded.
- Positive findings should be used for documentation only and counselling of the patients for further investigation given that they are asymptomatic women.

#### Recommended diagnostic work-up of selected population

The following thorough, preferably non-invasive, high accuracy diagnostic work-up is recommended for (1) *all symptomatic patients* of reproductive age, sexually active, *belonging to “high risk” groups* for the presence of a female genital anomaly and (2) *any asymptomatic woman suspected* to have anomaly from routine evaluation and wishing to undergo a more thorough evaluation. Furthermore, although they could not be considered as symptomatic, careful inspection is recommended for infertile patients after a first trimester miscarriage where foetal heart beats and for those entering IVF and/or older than 35 years old.

- Gynaecological examination with carefull evaluation and recording of the external genitalia, vaginal and cervical anatomy.
- 2D US (vaginal) in a pre-defined and systematic manner (to increase its diagnostic accuracy), where the shape and the dimensions of the uterine cavity, the uterine wall (anterior, posterior, lateral and fundal width) and external uterine contour should be recorded in a systematic way and pre-defined way in longitudinal and transverse planes. Measurements of 2D US examination should be used as a referendum for the evaluation of uterine anatomy deviations in 3D ultrasound.
- 3D US (vaginal) in a pre-defined and systematic manner where the shape and the deviations from normal cervical and uterine anatomy should be recorded and documented.

In subgroups of patients with *subfertility, recurrent IVF failures or recurrent pregnancy losses* additional examinations can be performed:

- HyCoSy or 2D or 3D SHG by an experienced sonographer when available.
- Hysteroscopy and, in cases of suspected adnexal pathology, hydrolaparoscopy or laparoscopy. Those techniques should be offered by clinicians, endoscopic reproductive surgeons, having also the ability to surgically treat any discovered pathology.
- X-ray HSG, nowadays, should not be considered anymore as a “first-line” diagnostic procedure and should be reserved only for settings where the pre-mentioned diagnostic methods are not available or for health systems where indicated for other reasons. Congenital uterine anomaly may be suspected from HSG performed in women with infertility to verify tubal patency”.

#### Recommended diagnostic work-up for complex anomalies

Sub-groups of patients with suspected *complex anomalies* (defined as anomalies resulting from disturbances in more than one stage of normal embryological development and having as a result anatomical deviations in more than one organ of the female genital tract) and *those where the application of the previously mentioned methods could not be applied* (e.g. obstructing anomalies) should be evaluated as follows:

- Abdominal and/or transrectal 3D US in a pre-defined and systematic manner where the shape and the deviations from normal cervical and uterine anatomy should be recorded and documented.
- MRI: evaluation of the results is recommended to be done by an imaging expert in collaboration with an experienced gynaecologist.
- Hysteroscopy and laparoscopy: these techniques should be offered by clinicians (endoscopic reproductive) and surgeons with experience in the management of complex female genital anomalies in special centres after thorough non-invasive evaluation and, mainly, in the context of concomitant surgical treatment of any discovered pathology.

#### Recommended diagnostic work-up for adolescents

*Adolescents with symptoms suggestive* for the presence of a female genital anomaly (primary amenorrhea and/or pelvic masses or pathology and/or cyclic pelvic pain) should be evaluated as follows:

- Gynaecological examination with careful evaluation and recording of the external genitalia.
- Abdominal and/or transrectal 2D US where the presence, the shape and the dimensions of the uterus (cavity, wall and external contour) should be recorded in a systematic and pre-defined manner in longitudinal and transverse planes.
- Abdominal and/or transrectal 3D US where the shape and the deviations from normal cervical and uterine anatomy should be recorded and documented.
- MRI as a first-line diagnostic procedure. Evaluation of the results is recommended to be done by an imaging expert in collaboration with an experienced gynaecologist.
- Hysteroscopy and laparoscopy: those techniques should be offered in the context of concomitant surgical treatment of any discovered pathology and only by endoscopic reproductive surgeons with experience in the management of complex female genital anomalies in special centres after thorough non-invasive evaluation.

In patients with female genital anomalies, *investigation of the urinary tract* is also recommended as mandatory.

#### Conclusion

The combination of gynaecological examination and 2D US could be recommended as the current standard for the evaluation of asymptomatic women; 3D US could be considered the standard for diagnosis of female genital anomalies supplemented by MRI, hysteroscopy and laparoscopy in complex ones or in diagnostic dilemmas.

#### Open issues for further research

The role of a combined ultrasound examination together with outpatient hysteroscopy as a one-stop diagnostic evaluation of symptomatic “high-risk” patients should be prospectively evaluated. The ESHRE/ESGE classification should be considered as a guide for diagnosis offering a common terminology among the clinicians to convey the exact anatomical status of the female genital tract [29, 16]; based on that, it is a challenge for further research to objectively estimate the clinical consequences related to various degrees of uterine deformity, e.g. the length of the septum and the potential co-factors that are associated with poor reproductive outcome. Large prospective studies with correct classifications and accurate measurements of the length of midline indentations are needed to establish optimal indications of reconstructive surgery in patients with congenital uterine anomalies.

## References

[1] American Fertility Society (1988). The AFS classification of adnexal aghesions, distul tubal occlusion, tubal occlusion secondary to tubal ligation, tubal pregnancies, Mullerian anomalies and intrauterine adhesions. Fertil Steril.

[2] Alatas C, Aksoy E, Akarsu C, Yakin K, Aksoy S, Hayran M (1997). Evaluation of intrauterine abnormalities in infertile patients by sonohysterography. Hum Reprod.

[3] Alborzi S, Dehbashi S, Khodaee R (2003). Sonohysterosalpingographic screening for infertile patients. Int J Gynaecol Obstet.

[4] Altman DG (1991). Practical statistics for medical research.

[5] Bermejo C, Ten Martínez P, Cantarero R, Diaz D, Pérez Pedregosa J, Barrón E, Labrador E, Ruiz López L (2010). Three-dimensional ultrasound in the diagnosis of Müllerian duct anomalies and concordance with magnetic resonance imaging. Ultrasound Obstet Gynecol.

[6] Bocca SM, Oehninger S, Stadtmauer L, Agard J, Duran EH, Sarhan A, Horton S, Abuhamad AZ (2012). A study of the cost, accuracy, and benefits of 3-dimensional sonography compared with hysterosalpingography in women with uterine abnormalities. J Ultrasound Med.

[7] Brown SE, Coddington CC, Schnorr J, Toner JP, Gibbons W, Oehninger S (2000). Evaluation of outpatient hysteroscopy, saline infusion hysterosonography, and hysterosalpingography in infertile women: a prospective, randomized study. Fertil Steril.

[8] Brucker SY, Rall K, Campo R, Oppelt P, Isaacson K (2011). Treatment of congenital malformations. Semin Reprod Med.

[9] Buttram VC, Gibbons WE (1979). Mullerian anomalies: a proposed classification (an analysis of 144 cases). Fertil Steril.

[10] Carrington BM, Hricak H, Nuruddin RN, Secaf E, Laros RK, Hill EC (1990). Müllerian duct anomalies: MR imaging evaluation. Radiology.

[11] Chan YY, Jayaprakasan K, Zamora J, Thornton JG, Raine-Fenning N, Coomarasamy A (2011). The prevalence of congenital uterine anomalies in unselected and high-risk populations: a systematic review. Hum Reprod Update.

[12] Chan YY, Jayarpakasan K, Tan A, Thornton JG, Coomarasamy A, Raine-Fenning NJ (2011). Reproductive outcomes in women with congenital uterine anomalies: a systematic review. Ultrasound Obstet Gynecol.

[13] Console D, Tamburrini S, Barresi D, Notarangelo L, Bertucci B, Tamburrini O (2001). The value of the MR imaging in the evaluation of Müllerian duct anomalies. Radiol Med.

[14] De Felice C, Porfiri LM, Savelli S, Alfano G, Pace S, Manganaro L, Vestri AR, Drudi FM (2009). Infertility in women: combined sonohysterography and hysterosalpingography in the evaluation of the uterine cavity. Ultraschall Med.

[15] Deutch T, Bocca S, Oehninger S (2006). Magnetic resonance imaging versus three-dimensional transvaginal ultrasound for the diagnosis of Müllerian anomalies. Fertil Steril.

[16] Di Spiezio Sardo A, Campo R, Gordts S, Spinelli M, Cosimato C, Tanos V, Brucker S, Li TC, Gergolet M, De Angelis C, Gianaroli L, Grimbizis G (2015). The comprehensiveness of the ESHRE/ESGE classification of female genital tract congenital anomalies: a systematic review of cases not classified by the AFS system. Hum Reprod.

[17] Dodero D, Corticelli A, Caporale E, Cardamone C, Francescangeli E (2001). Benign uterine pathology in premenopause and transvaginal sonohysterography: personal experience. Minerva Ginecol.

[18] Faivre E, Fernandez H, Deffieux X, Gervaise A, Frydman R, Levaillant JM (2012). Accuracy of three-dimensional ultrasonography in differential diagnosis of septate and bicornuate uterus compared with office hysteroscopy and pelvic magnetic resonance imaging. J Minim Invasive Gynecol.

[19] Fedele L, Dorta M, Brioschi D, Massari C, Candiani GB (1989). Magnetic resonance evaluation of double uteri. Obstet Gynecol.

[20] Fedele L, Bianchi S, Zanconato G, Berlanda N, Bergamini (2005). Laparoscopic removal of the cavitated noncommunicating rudimentary uterine horn: surgical aspects in 10 cases. Fertil Steril.

[21] Gergolet M, Rudi Campo R, Verdenik I, Kenda Suster N, Gordts S, Gianaroli L (2012). No clinical relevance of the height of fundal indentation in subseptate or arcuate uterus: a prospective study. Reprod Biomed Online.

[22] Ghi T, Casadio P, Kuleva M, Perrone AM, Savelli L, Giunchi S, Meriggiola MC, Gubbini G, Pilu G, Pelusi C, Pelusi G (2009). Accuracy of three-dimensional ultrasound in diagnosis and classification of congenital uterine anomalies. Fertil Steril.

[23] Goldberg JM, Falcone T, Attaran M (1997). Sonohysterographic evaluation of uterine abnormalities noted on hysterosalpingography. Hum Reprod.

[24] Grimbizis GF, Camus M, Tarlatzis BC, Bontis JN, Devroey P (2001). Clinical implications of uterine malformations and hysteroscopic treatment results. Hum Reprod Update.

[25] Grimbizis GF, Tsalikis T, Mikos T, Papadopoulos N, Tarlatzis BC, Bontis JN (2004). Successful end-to-end cervico-cervical anastomosis in a patient with congenital cervical fragmentation: case report. Hum Reprod.

[26] Grimbizis GF, Campo R (2010). Congenital malformations of the female genital tract: the need for a new classification system. Fertil Steril.

[27] Grimbizis GF, Gordts G, Di Spiezio SA, Brucker S, De Angelis C, Gergolet M, Li T-C, Tanos V, Brölmann H, Gianaroli L, Campo R (2013). The ESHRE/ESGE consensus on the classification of female genital tract congenital malformations. Hum Reprod.

[28] Grimbizis GF, Gordts G, Di Spiezio SA, Brucker S, De Angelis C, Gergolet M, Li T-C, Tanos V, Brölmann H, Gianaroli L, Campo R (2013). The ESHRE/ESGE consensus on the classification of female genital tract congenital malformations. Gynecol Surg.

[29] Grimbizis GF, Gordts G, Di Spiezio SA, Brucker S, De Angelis C, Gergolet M, Li T-C, Tanos V, Brölmann H, Gianaroli L, Campo R (2014). Reply: are the ESHRE/ESGE criteria of female genital anomalies for diagnosis of septate uterus appropriate?. Hum Reprod.

[30] Guimaraes Filho HA, Mattar R, Pires CR, Araujo Junior E, Moron AF, Nardozza LM (2006). Comparison of hysterosalpingography, hysterosonography and hysteroscopy in evaluation of the uterine cavity in patients with recurrent pregnancy losses. Arch Gynecol Obstet.

[31] Imboden S, Müller M, Raio L, Mueller MD, Tutschek B (2014). Clinical significance of 3D ultrasound compared to MRI in uterine malformations. Ultraschall Med.

[32] Joki-Erkkilä MM, Heinonen PK (2003). Presenting and long-term clinical implications and fecundity in females with obstructing vaginal malformations. J Pediatr Adolesc Gynecol.

[33] Jones J, Hunter D (1995). Consensus methods for medical and health services research. BMJ.

[34] Keltz MD, Olive DL, Kim AH, Arici A (1997). Sonohysterography for screening in recurrent pregnancy loss. Fertil Steril.

[35] Laganà AS, Ciancimino L, Mancuso A, Chiofalo B, Rizzo P, Triolo O (2014). 3D sonohysterography vs hysteroscopy: a cross-sectional study for the evaluation of endouterine diseases. Arch Gynecol Obstet.

[36] Letterie GS, Haggerty M, Lindee G (1995). A comparison of pelvic ultrasound and magnetic resonance imaging as diagnostic studies for Müllerian tract abnormalities. Int J Fertil Menopausal Stud.

[37] Ludwin A, Ludwin I, Banas T, Knafel A, Miedzyblocki M, Basta A (2011). Diagnostic accuracy of sonohysterography, hysterosalpingography and diagnostic hysteroscopy in diagnosis of arcuate, septate and bicornuate uterus. J Obstet Gynaecol Res.

[38] Ludwin A, Pityński K, Ludwin I, Banas T, Knafel A (2013). Two- and three-dimensional ultrasonography and sonohysterography versus hysteroscopy with laparoscopy in the differential diagnosis of septate, bicornuate, and arcuate uteri. J Minim Invasive Gynecol.

[39] Makris N, Kalmantis K, Skartados N, Papadimitriou A, Mantzaris G, Antsaklis A (2007). Three-dimensional hysterosonography versus hysteroscopy for the detection of intracavitary uterine abnormalities. Int J Gynaecol Obstet.

[40] Marten K, Vosshenrich R, Funke M, Obenauer S, Baum F, Grabbe E (2003). MRI in the evaluation of Müllerian duct anomalies. Clin Imaging.

[41] Minto CL, Hollings N, Hall-Craggs M, Creighton S (2001). Magnetic resonance imaging in the assessment of complex Müllerian anomalies. BJOG.

[42] Moini A, Mohammadi S, Hosseini R, Eslami B, Ahmadi F (2013). Accuracy of 3-dimensional sonography for diagnosis and classification of congenital uterine anomalies. J Ultrasound Med.

[43] Mollo A, De Franciscis P, Colacurci N, Cobellis L, Perino A, Venezia R, Alviggi C, De Placido G (2009). Hysteroscopic resection of the septum improves the pregnancy rate of women with unexplained infertility: a prospective controlled trial. Fertil Steril.

[44] Momtaz MM, Ebrashy AN, Marzouk AA (2007). Three-dimensional ultrasonography in the evaluation of the uterine cavity. Middle East Fertil Soc J.

[45] Mueller GC, Hussain HK, Smith YR, Quint EH, Carlos RC, Johnson TD, DeLancey JO (2007). Müllerian duct anomalies: comparison of MRI diagnosis and clinical diagnosis. AJR Am J Roentgenol.

[46] Nicolini U, Bellotti M, Bonazzi B, Zamberletti D, Candiani GB (1987). Can ultrasound be used to screen uterine malformations?. Fertil Steril.

[47] Pellerito JS, McCarthy SM, Doyle MB, Glickman MG, DeCherney AH (1992). Diagnosis of uterine anomalies: relative accuracy of MR imaging, endovaginal ultrasound, and hysterosalpingography. Radiology.

[48] Preutthipan S, Linasmita V (2003). A prospective comparative study between hysterosalpingography and hysteroscopy in the detection of intrauterine pathology in patients with infertility. J Obstet Gynaecol Res.

[49] Radoncic E, Funduk-Kurjak B (2000). Three-dimensional ultrasound for routine check-up in in vitro fertilization patients. Croat Med J.

[50] Ragni G, Diaferia D, Vegetti W, Colombo M, Arnoldi M, Crosignani PG (2005). Effectiveness of sonohysterography in infertile patient work-up: a comparison with transvaginal ultrasonography and hysteroscopy. Gynecol Obstet Investig.

[51] Raziel A, Arieli S, Bukovsky I, Caspi E, Golan A (1994). Investigation of the uterine cavity in recurrent aborters. Fertil Steril.

[52] Rock JA, Roberts CP, Jones HW (2010). Congenital anomalies of the uterine cervix: lessons from 30 cases managed clinically by a common protocol. Fertil Steril.

[53] Santos XM, Krishnamurthy R, Bercaw-Pratt JL, Dietrich JE (2012). The utility of ultrasound and magnetic resonance imaging versus surgery for the characterization of Müllerian anomalies in the pediatric and adolescent population. J Pediatr Adolesc Gynecol.

[54] Saravelos SH, Cocksedge KA, Li T-C (2008). Prevalence and diagnosis of congenital uterine anomalies in women with reproductive failure: a critical appraisal. Hum Reprod Update.

[55] Soares SR, Barbosa dos Reis MM, Camargos AF (2000). Diagnostic accuracy of sonohysterography, transvaginal sonography, and hysterosalpingography in patients with uterine cavity diseases. Fertil Steril.

[56] Strawbrigde LC, Crough NS, Cutner AS, Creighton SM (2007). Obstructive Mullerian anomalies and modern laparoscopic management. J Pediatr Adolesc Gynecol.

[57] Traina E, Mattar R, Moron AF, Neto LCA, Matheus EDE (2004). Diagnostic accuracy of hysterosalpingography and transvaginal sonography to evaluate uterine cavity diseases in patients with recurrent miscarriage. Rev Bras Ginecol Obstet.

[58] Valenzano MM, Mistrangelo E, Lijoi D, Fortunato T, Lantieri PB, Risoo D, Constantini S, Ragni N (2006). Transvaginal sonohysterographic evaluation of uterine malformations. Eur J Obstet Gynecol Reprod Biol.

[59] Venetis C, Papadopoulos S, Campo R, Gordts S, Tarlatzis BC, Grimbizis GF (2014). Clinical implications of congenital uterine anomalies: a meta-analysis of comparative studies. Reprod Biomed Online.

[60] Wu MH, Hsu CC, Huang KE (1997). Detection of congenital Müllerian duct anomalies using three-dimensional ultrasound. J Clin Ultrasound.

